# Synovial Sarcoma Arising From the Falciform Ligament

**DOI:** 10.7759/cureus.99576

**Published:** 2025-12-18

**Authors:** Amelia J Cooper, Magdalena Sejka

**Affiliations:** 1 General Surgery, Eastern Health, Melbourne, AUS; 2 General Surgery, Albury Wodonga Health, Albury, AUS

**Keywords:** abdominal synovial sarcoma, falciform ligament, falciform ligament malignancy, soft tissue sarcoma, synovial cell sarcoma

## Abstract

Synovial sarcoma is a rare soft tissue malignancy that is most commonly found in the extremities of adolescents and young adults. Despite accounting for a small percentage of all soft tissue sarcomas, its aggressive behaviour makes early recognition and management crucial. We present the rare case of a 64-year-old man with a large epigastric mass extending into the mediastinum and involving the pericardium, which was found to be a synovial sarcoma arising from the falciform ligament. The tumour was locally invading the liver and anterior mediastinum and was associated with pulmonary metastases. Despite treatment with neoadjuvant chemotherapy, the tumour demonstrated only a partial response at the primary site and progression of metastatic disease, precluding surgical resection.

## Introduction

Synovial sarcoma is a rare form of soft tissue sarcoma that is typically diagnosed in the extremities of adolescents and young adults [1]. Despite the name, it is now known that synovial sarcoma does not originate from synovial tissue but rather from mesenchymal cells capable of epithelial differentiation [2]. Histologically, synovial sarcoma is classified into three subtypes: monophasic, biphasic, and poorly differentiated. The poorly differentiated variant is associated with more aggressive behaviour and worse prognosis [3].

Although synovial sarcoma is primarily found in the limbs, it has also been reported in other locations, including the mediastinum and abdomen. These non-extremity presentations are rare and associated with delayed diagnosis, limited options for resection, and poorer outcomes [4,5]. This case report describes what appears to be the first documented case of synovial sarcoma arising from the falciform ligament. It contributes to the limited literature on non-extremity synovial sarcomas and highlights the need for further research into systemic treatment options beyond current standards.

## Case presentation

A 64-year-old man presented to a regional emergency department complaining of two days of constant epigastric pain and anorexia. He denied changes in bowel habit, dysphagia, loss of weight and shortness of breath. He had no significant medical history and took no regular medications.

Examination revealed a fit man with a mildly elevated temperature of 37.8°C and a tender epigastrium with a palpable, firm, immobile mass. C-reactive protein (CRP) was raised at 272 mg/L (normal range: 0-10 mg/L), and bilirubin was mildly raised at 35 μmol/L (normal range: 0-20 μmol/L). Otherwise, biochemistry and tumour markers, including carcinoembryonic antigen (CEA), cancer antigen 19-9 (CA 19-9), cancer antigen 13-3 (CA 15-3) and alpha-fetoprotein, were unremarkable.

**Figure 1:**
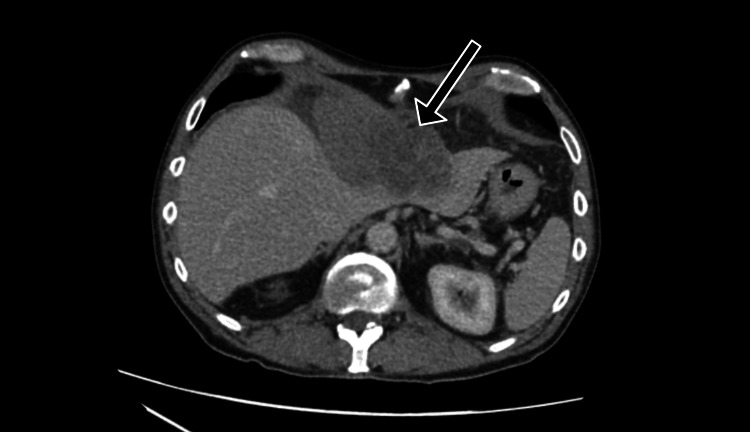
Axial portal venous phase CT of the abdomen showing a large epigastric mass with evidence of local invasion into the liver.

**Figure 2:**
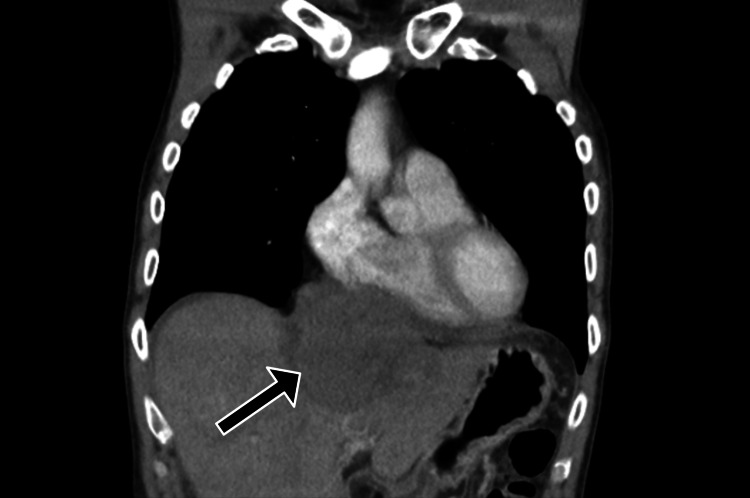
Coronal chest slice from a portal venous phase CT demonstrating a large epigastric mass with local invasion into the liver and pericardial thickening, raising concern for possible pericardial involvement.

A contrast-enhanced computed tomography (CT) scan of the chest, abdomen, and pelvis revealed a large 10.2 x 5.5 x 9.6 cm epigastric mass mostly exophytic to the liver, seeming to arise from the parenchyma of the left lobe of the liver (Figure 1). The mass extended approximately 60 mm below the xiphisternum and superiorly extended into the thoracic cavity and became closely associated with the pericardium, with thickening, raising the possibility of direct pericardial involvement. On further evaluation, it was deemed to be arising from the falciform ligament and invading into the left lobe of the liver as well as into the anterior mediastinum through the diaphragm (Figure 2). There was also a markedly enlarged pericardiac lymph node with a maximal dimension of 25 mm.

Further CT imaging of the head, neck, and chest revealed multiple pulmonary nodules, suggesting pulmonary metastasis.

**Figure 3:**
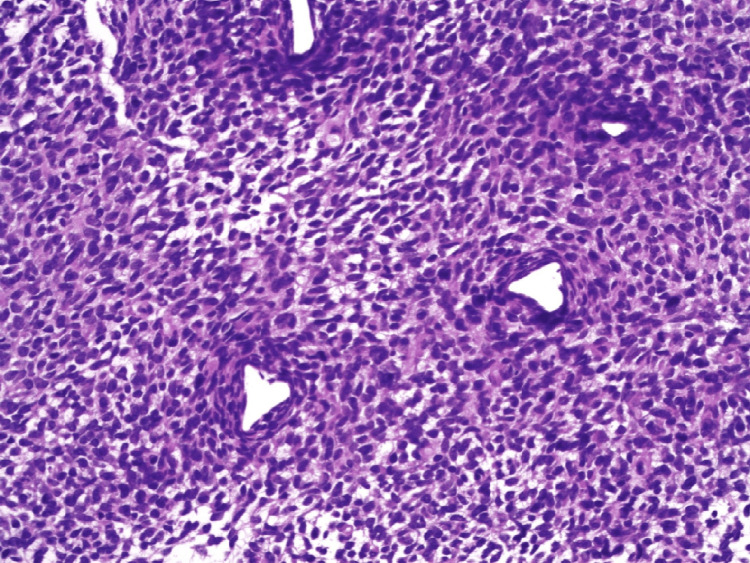
Haematoxylin and eosin-stained section demonstrating poorly differentiated synovial sarcoma.

An ultrasound-guided core biopsy was performed, and histology initially showed an undifferentiated malignancy characterised by poorly differentiated sheets of cells with hyperchromatic spindle nuclei and moderate cytoplasm (Figure 3). Further evaluation with fluorescence in situ hybridisation (FISH) panel showed SS18-SSX2 fusion, which favoured a diagnosis of synovial sarcoma. On subsequent review of the histopathology, the tumour was classified as a poorly differentiated synovial sarcoma.

The patient was sent to a specialty cancer centre, where he underwent neoadjuvant chemotherapy with doxorubicin and ifosfamide with the intent for curative resection. However, the patient had a poor response to chemotherapy, and despite some response in size and fluorodeoxyglucose (FDG)-avidity of the primary tumour, there was progression of metastases in the mediastinum and lungs. Given the progression of disease while on neoadjuvant chemotherapy, the decision was made to proceed with palliative pazopanib therapy.

## Discussion

The name 'synovial sarcoma' arose during the time when it was thought that these tumours developed from synovial cells, given their predilection for joints and other structures in the limbs. However, it is now believed that synovial sarcomas arise from pluripotent mesenchymal cells that are capable of differentiating into epithelial cells [1] and SS18-SSX1, SS18-SSX2, or SS18-SSX4 fusion oncoproteins [2]. The falciform ligament is a double layer of peritoneum that contains mesenchyme-derived loose connective tissue, small vessels, lymphatics, and variable amounts of fat [6]. It is this connective tissue, which embryologically arises from the mesoderm, that can give rise to synovial sarcoma, as in this case.

Synovial sarcoma can be divided into three histological subtypes: monophasic, biphasic, and poorly differentiated. The monophasic type contains spindle cells without a glandular component, while the biphasic type contains a combination of spindle cells and epithelial cells [3]. Poorly differentiated synovial sarcoma, as in this case, is the most aggressive subtype and typically exhibits sheets of small, round cells with high levels of mitotic activity [3]. CRP elevation in synovial sarcoma is typically driven by tumour-associated inflammation and cytokine release, particularly IL-6, and may be exacerbated by tumour necrosis or secondary infection [7].

Survival outcomes in synovial sarcoma vary substantially. Reported five-year survival rates range between 55% and 75% [8], while patients with metastatic disease face a much poorer outlook, with approximately 10% surviving to 10 years [9]. The behaviour of synovial sarcomas is heterogeneous and depends on histological subtype, making prognostic predictions difficult [4]. The prognosis is reported to be adversely affected by increased age at diagnosis, the central location of the tumour, the stage at presentation, increased tumour size, and male sex [8]. Upper limb tumours have been found to have a better prognosis than lower limb tumours, and non-extremity tumours have the worst prognosis [4].

The poor prognosis of synovial sarcomas is due to the highly aggressive nature of these tumours. Surgical resection is the basis of treatment and is often combined with chemotherapy or radiotherapy [1]. First-line systemic treatment for metastatic synovial sarcoma is with anthracyclines (e.g., doxorubicin) and ifosfamide; however, response rates remain low, with less than 30% [10]. A standard second-line therapy has not been established, but pazopanib and trabectedin are often used [10].

There have been reports of large mediastinal synovial sarcomas responding well to neoadjuvant chemotherapy with doxorubicin and ifosfamide, leading to complete surgical resection and no sign of recurrence after five years [11]. Notably, however, the patient in that case report had no evidence of distant metastases. Pulmonary metastases are a major cause of mortality in patients with soft tissue sarcomas, and successful resection of these metastases has been found to positively influence survival [12].

While synovial sarcoma is most often found in the extremities, there have been reports of this tumour arising from the chest wall, lung, and mediastinum [1,5] as well as from the heart itself [12]. The survival rate of synovial sarcomas has remained stagnant over the past few decades, owing to a lack of progress in management strategies [4]. In addition, there is a paucity of research regarding the prognosis and treatment of synovial sarcomas specifically arising from areas other than limbs.

## Conclusions

This report describes the presence of synovial sarcoma formation in the falciform ligament, a highly uncommon anatomical location for this tumour type. In this case, the patient presented with a synovial sarcoma of the chest and upper abdomen, which was initially thought to be resectable after neoadjuvant chemotherapy. Diagnostic challenges are highlighted in this case, given the rarity of this tumour type in the abdomen. Given that synovial sarcoma is typically found in the extremities, non-extremity presentations risk under-recognition, emphasising the need for awareness of atypical sites.

Additionally, synovial sarcoma can appear non-specific radiologically; therefore, biopsy remains crucial to direct management and avoid misclassification. A poor response of the tumour in this case to the current best treatment, doxorubicin and ifosfamide, led to advancing metastasis and an unresectable tumour. This case illustrates the need for further research into other systemic therapies available for this rare but aggressive tumour, where complete resection is the gold standard of treatment.
